# Major sources of critical incidents in intensive care

**DOI:** 10.1186/cc10474

**Published:** 2011-09-29

**Authors:** Ingeborg D Welters, James Gibson, Martin Mogk, Richard Wenstone

**Affiliations:** 1 Intensive Care Unit, Royal Liverpool University Hospital, Prescot Street, Liverpool, L7 8XP, UK; 2 Institute of Ageing and Chronic Disease, University of Liverpool, The Duncan Building, Daulby Street, Liverpool, L69 3GA, UK; 3 Moredata GmbH, Kerkrader Str. 11, D-35394 Giessen, Germany

## Abstract

**Introduction:**

In recent years, critical incident (CI) reporting has increasingly been regarded as part of ongoing quality management. CI databanks also aim to improve health and safety issues for patients as well as staff. The aim of this study was to identify frequent causes of adverse events in critical care with the potential to harm patients, staff or visitors by analysing data from a voluntary and optionally anonymous critical incident reporting system.

**Methods:**

The study includes all critical incidents reported during a 90-month period in a 13-bed adult general intensive care unit (ICU). Reporting of incidents was performed via an electronic reporting system or by a manual critical incident report. All CIs were classified in the following main categories: equipment, administration, pharmaceuticals, clinical practice, and health & safety hazards. The overall distribution of incidents within the different categories was compared with the regional database of ICUs in the Cheshire and Mersey region of northwest England for 2008.

**Results:**

A total of 1127 CIs were reported during the study period. The frequencies within the main categories were: equipment 338 (30%), clinical practice 257 (22.8%), pharmaceuticals 238 (21.1%), administration 213 (18.9%), health and safety hazards 81 (7.2%). The regional database had a similar frequency of critical incidents within the different categories, suggesting that our results may reflect a general distribution pattern of CIs in intensive care.

**Conclusions:**

Critical incident reporting helps to identify frequent causes of adverse events in critical care. Improvements in quality of care following implementation of preventative strategies such as introduction of regular equipment training sessions will have to be assessed further in future studies.

## Introduction

Several circumstances predispose the critically ill patient to medical errors and adverse events. The patient in intensive care will be exposed to a higher number of invasive procedures, numerous medical devices, and the risks associated with polypharmacy. Several studies and reviews have highlighted that medication errors represent a high percentage of critical incidents (CIs) reported in critically ill patients [1-3]. In addition, intensive care unit (ICU) patients are more likely to experience treatment- or procedure-related complications such as equipment failure, unplanned dislodgement or inappropriate disconnection of lines, catheters, or drains, and errors related to medication or airway complications [4]. It is not surprising that many studies report a higher incidence of adverse events in the ICU than in other health-care settings. Although the number of publications on CI reporting has risen considerably during the last few years, very few results from large databases with several years of reporting are available [5,6]. Our aim was to identify the most frequent types of CIs in intensive care and, if possible, identify any recurring pattern of errors occurring over the course of a 90-month period. Thus, we conducted a prospective, observational, single-center study on the frequency and characteristics of CIs in a 13-bed general ICU at a university hospital. In addition, we compared the frequency of each class of CIs in our ICU with data obtained from the regional critical care network database.

## Materials and methods

Ethical approval was not required since this project is classified by the National Ethics Research Service 'Defining Research' guidance as a service evaluation.

### Data collection

In a closed, multi-disciplinary, adult, 13-bed ICU, a new paper-based CI form was introduced in July 2002. This comprised a single page with most of the space allowing free text. The form contained general information such as date and time as well as space for a narrative of the incident. No attempt was made to direct the reporter to any type of classification system either of the incident or of its seriousness. The reporter could choose to remain anonymous if they wished. The introduction of this form was preceded by an educational drive to encourage reporting of anything that was felt by staff to pose or potentially pose a hazard to staff, patients, or visitors. Staff members were still free to report on the traditional, hospital-wide paper forms throughout this time instead of, or in addition to, the new ICU-specific form.

From late 2000, the hospital began to introduce an electronic reporting system (Datix Ltd, London, UK), although this did not become accessible to ICU staff until late 2005, at which point the hospital's paper form was withdrawn. The electronic reporting system is a context-driven menu-based system of pick lists plus free-text boxes, all of which are customized by the hospital. The reporter is directed to classify each incident in terms of severity and likelihood of recurrence. The process does not allow anonymity. Completed electronic forms are automatically e-mailed to appropriate clinical and managerial staff within and outside the ICU (depending on the location and nature of the incident) as well as to the hospital's risk manager. These staff members are then responsible for the investigation and, if necessary, re-classification of the type or severity of each incident (or both). However, with the aim of trying to maintain high reporting rates, the ICU-specific paper form was retained, enabling staff to use either or both of the reporting mechanisms.

All reports (that is, hospital paper-based plus ICU-specific paper-based from July 2002 to November 2005 and hospital electronic plus ICU-specific paper-based from December 2005 to the present time) were entered into a datasheet. (Microsoft^®, ^Excel; Microsoft Corporation, Redmond, WA, USA). Feedback was provided to ICU staff by a combination of monthly meetings, e-mail to all staff, and a notice board within the ICU.

### Incident analysis

Similarly to investigators in previous studies [7,8], we classified pharmaceutical incidents and equipment-related incidents. Overall, we defined five categories - equipment, clinical practice, administration, pharmaceuticals, and health and safety hazards - for all electronic and paper reports. Examples of equipment-related incidents include dislodgement and disconnection of lines and tubes, unfamiliarity with equipment, faulty equipment, and unavailability of required devices. The majority of incidents reported under 'clinical practice' were pressure sores, and events related to handling of specimens, infection control, and airway management. Shortages in staffing, insufficient availability of critical care beds, and shortcomings in documentation and identification of patients were summarized in 'administration'. The category 'pharmaceuticals' contained errors in prescription, administration of the wrong dose or drug, the wrong route of administration, and labeling, storage, and availability of prescribed drugs. 'Health and safety hazards' referred to incidents such as slips, trips, and falls and other injuries to staff and environmental risks. Where there were difficulties in assigning the incidents to a category, consensus was reached after discussion between three of the authors (RW, IDW, and JG).

### Comparison with regional data

To explore whether the frequency and type of CIs reported in our ICU followed a more general pattern, all CIs documented in 2008 were compared with the regional database. In 2007, the regional Critical Care Network comprising our ICU and 10 other ICUs in our geographical area (Cheshire and Merseyside) in northwest England started to collect CI data from each of its ICUs. These other units, which collected data in 2008, represented 143 critical care beds in total. Thus, it was possible to compare the 2008 data set of our ICU with that of neighboring ICUs with respect to types and frequencies of various CIs. Three of the authors (RW, IDW, and JG) classified all data from the regional database into the same categories used for our data.

### Statistical analysis

Descriptive statistics, including frequencies and distribution within each category, were performed and displayed as graphs, figures, and tables as appropriate. Percentages of the total reported incidents were calculated for each category. Chi-square tests were performed to assess differences between regional and local frequencies of CIs.

## Results

### Distribution of critical incidents

**Figure 1:**
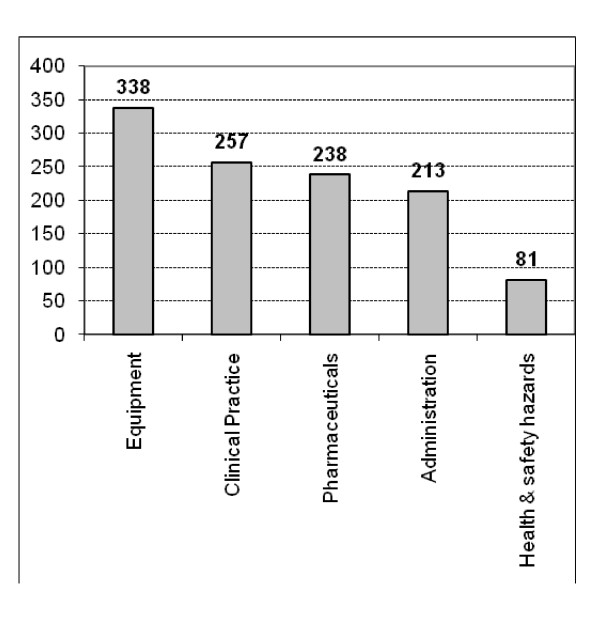
**Distribution of critical incidents within five main categories**. A total of 1,127 critical incidents reported in a 90-month period in our intensive care unit were analyzed.

**Figure 2:**
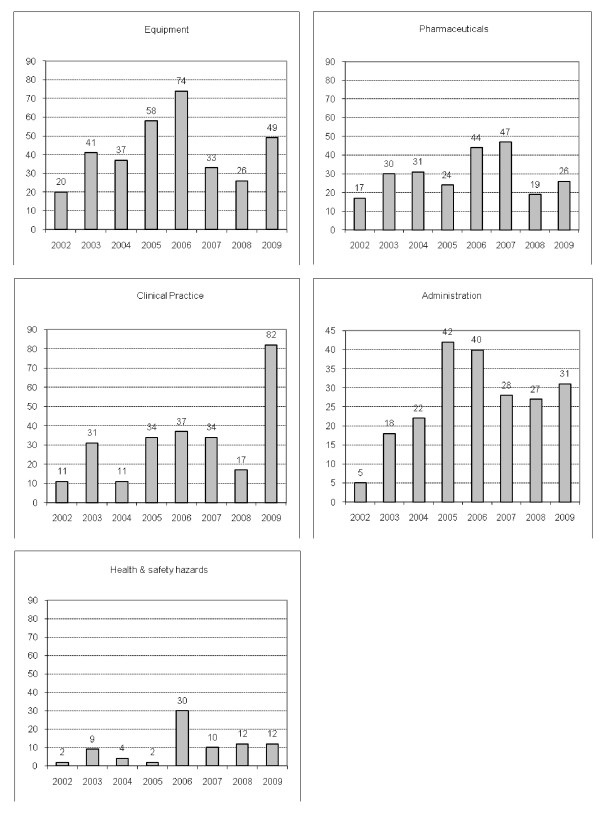
**Distribution of critical incidents by year for each of five main categories**. Values refer to incidents reported in our intensive care unit during the study period.

A total of 1,127 CIs were reported during the study period in our ICU. The frequencies of CIs within the main categories are shown in Figure 1. CIs involving equipment represented the main fraction (30.0%); however, CIs in clinical practice (22.8%) and those involving drugs and pharmaceuticals (21.1%) as well as administration-related incidents (18.9%) were also common. There was no trend toward higher reporting in any of the CI classes during the observation period (Figure 2 and Table 1).

**Table 1:** Distribution of critical incidents by category and year

Category Category	2002	2003	2004	2005	2006	2007	2008	2009	Total
Equipment	20 (1.8)	41 (3.6)	37 (3.3)	58 (5.1)	74 (6.6)	33 (2.9)	26 (2.3)	49 (4.3)	338 (30.0)
Clinical practice	11 (1.0)	31 (2.8)	11 (1.0)	34 (3.0)	37 (3.3)	34 (3.0)	17 (1.5)	82 (7.3)	257 (22.8)
Pharmaceuticals	17 (1.5)	30 (2.7)	31 (2.8)	24 (2.1)	44 (3.9)	47 (4.2)	19 (1.7)	26 (2.3)	238 (21.1)
Administration	5 (0.4)	18 (1.6)	22 (2.0)	42 (3.7)	40 (3.5)	28 (2.5)	27 (2.4)	31 (2.9)	213 (18.9)
Health and safety hazards	2 (0.2)	9 (0.8)	4 (0.4)	2 (0.2)	30 (2.7)	10 (0.9)	12 (1.1)	12 (1.1)	81 (7.2)
Total	55 (4.9)	129 (11.4)	105 (9.3)	160 (14.2)	225 (20.0)	152 (13.5)	101 (9.0)	200 (17.7)	1,127 (100)

Values refer to incidents reported in our intensive care unit during the study period.

Equipment-related incidents were the most frequently reported (a total of 338 incidents). The most common equipment incident was faulty equipment, which accounted for 113 CIs (33.4%); unfamiliarity or incorrect use of equipment accounted for 72 CIs (21.3%); disconnection and leaks accounted for 65 CIs (19.2%); and lack of equipment or unavailability of equipment accounted for 64 CIs (18.9%). The remaining 24 (7.1%) equipment-related incidents arose from 20 other subcategories.

Two hundred thirty-eight pharmaceutical incidents were reported. Administration of an incorrect dose, the commonest incident, accounted for 61 (25.6%) incident reports. There were 60 (25.2%) reports of lack of availability of a drug. Errors in administration of a drug, including incorrect rate or route, were reported 25 (10.5%) times, and prescription errors were found in 21 (8.8%) reports. In 38 (16.0%) cases, the wrong drug was administered. The remaining 33 (13.9%) CIs related to pharmaceuticals included errors in drug labeling (13 incidents), the recording or handling of controlled drugs, blood transfusions, and storage of pharmaceuticals.

Clinical practice incidents, including those related to patient transfers, accounted for 257 incident reports. There were 114 (44.3%) reported pressure sores, change coma to semicolon 25 (9.7%) incidents related to the handling of clinical specimens, and there were 20 (7.8%) infection control incidents; this last group includes infections such as methicillin-resistant *Staphylococcus aureus *which are reported by national mandate. Thirty-four (13.2%) airway-related incidents were reported; this number does not include those reported as disconnections in the equipment category. There were 27 (10.5%) CIs summarized as poor practice. Further reports (23 CIs or 8.9%) included disposal of sharps, the practice of blood sugar monitoring, and medical complications and adverse events during investigation or interventions. CIs during transfers of patients contributed 14 (5.4%) reports.

Two hundred thirteen reported incidents related to the administration and management of the ICU: 54 (25.3%) incidents of staffing shortages and 76 (35.7%) incidents of bed shortages were recorded. Documentation errors accounted for 45 (21.1%), communication failure for 15 (7.0%), and patient identification issues for 12 (5.6%) incidents. The remaining reports referred to discharge and level of care, patient property, and availability of patient meals (11 CIs or 5.2%).

Health and safety hazards produced 81 (7.2%) reported incidents, and there were 46 (56.8%) reported incidents of injury to staff (mainly needle-stick injuries). Slips, trips, and falls were reported in 13 (16.0%) cases and included staff, patients, and visitors. Environmental hazards were identified in 20 (24.7%) reports and the other two incidents were related to visitors to the unit.

### Comparison with regional data

In addition to our ICU, 9 critical care units contributed data to a regional database representing 143 level 2 and 3 beds. Four hundred four CIs reported in the regional database in 2008 were analyzed. The local hospitals with ICUs that contributed to the regional database were (a) Leighton Hospital, Crewe; (b) Liverpool Heart & Chest Hospital, Liverpool; (c) Macclesfield District General Hospital, Macclesfield; (d) Southport & Formby District General Hospital, Southport; (e) University Hospital Aintree, Liverpool; (f) Walton Centre for Neurology and Neurosurgery, Liverpool; (g) Warrington Hospital, Warrington; (h) Whiston Hospital, Prescot; and (i) Wirral University Teaching Hospital, Arrowe Park, Wirral.

**Figure 3:**
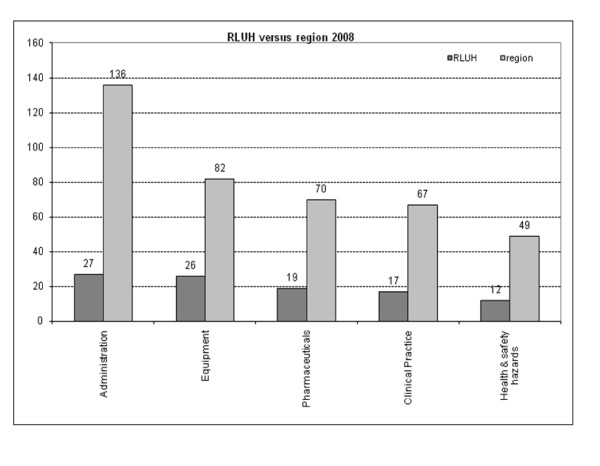
**Number of critical incidents reported at the Royal Liverpool University Hospital (RLUH) and throughout the North West Critical Care Network**. Exact numbers from 2008 data are specified in the graph.

On comparison with the regional CI database, a similar frequency of CIs within the different categories was noted (Figure 3 and Table 2), indicating that our results may reflect a general distribution pattern of CIs in intensive care. Interestingly, within the region, most of the reported incidents fell under 'administration', suggesting that the threshold for reporting administration-related incidents might be lower than for other types of CIs.

**Table 2:** Frequency of each category of critical incidents reported in our hospital and those reported in the region

	Percentage of total critical incidents in 2008		
Category	RLUH	Region	*P *value
Administration	26.8%	33.7%	0.183
Equipment	25.7%	20.3%	0.233
Pharmaceuticals	18.8%	17.3%	0.233
Clinical practice	16.8%	16.6%	0.726
Health and safety hazards	11.9%	12.1%	0.964
Total	100%	100%	

Mathematical deviations in sums are caused by rounding errors. Chi-square tests were performed on absolute numbers to assess differences between regional and local frequencies of critical incidents. RLUH, Royal Liverpool University Hospital.

## Discussion

The Institute of Medicine's 2000 report *To Err is Human *[9] showed that, in approximately 10% of hospital admissions, adverse events were described. Reporting CIs was described as a crucial factor in raising awareness about safety issues in medicine. Since then, incident reporting has been instituted in health-care systems in several countries. An effective incident reporting system facilitates early detection of factors related to causation and prevention of CIs in critical care areas. In addition, discussion of and feedback on CIs help to promote a culture of safety and learning [10]. A growing body of studies on CI reporting has been published in recent years, but only a few publications analyze large data sets [11] or longer observation periods. Many reports refer to CI reporting in anesthesia [12], but little evidence about the distribution of CIs in intensive care is available. Thus, the aim of our study was to identify the main areas in which CIs are regularly reported in intensive care. In our study, we analyzed 1,127 CIs reported over the course of a 90-month period after the introduction of a CI reporting system.

As part of our standard practice, all reported CIs were reviewed and discussed at monthly morbidity and mortality meetings to identify events that require immediate action. However, to establish frequency and recurrence of specific CIs, a longer observation period was required. In accordance with the hospital's policies, all CIs that led to major harm to patients or staff were investigated by root cause analysis. Amendment of guidelines and additional staff training sessions relating to the CI are examples of immediate -responses to CIs associated with severe patient harm. Specific CIs that triggered such changes include hypoglycemia associated with intravenous insulin administration and the dislodgement of central lines because of insufficient fixation.

The largest fraction (30%) of reported CIs was related to equipment, and one third of reports referred to faulty equipment (113 events). This is considerably lower than the percentage of equipment dysfunction reported to the National Patient Safety Agency: more than 50% of CIs associated with equipment were related to malfunction [13]. Dislodgement, disconnection, or leak of catheters and tubes as well as the unavailability of equipment were also common; 65 and 64 CIs, respectively, were reported. Incorrect handling (61) and unfamiliarity (11) were the second and third leading causes for reporting CIs related to equipment. In an attempt to address these equipment-related CIs, an equipment group was established to serve as a point of contact to discuss the purchase of new equipment as well as recurrent equipment faults and equipment-related safety concerns. In addition, training issues and handling errors were addressed by introducing a practice educator to follow up CIs related to these particular subgroups. The practice educator's role was to ensure the introduction of equipment to new staff as well as continuous education in the use and handling of all equipment available on the unit. After the appropriate training and an introduction to equipment, users' names were entered into a database against the relevant medical devices to record users' familiarity with them. Further studies to assess the change in equipment-related CIs after introduction of the practice educator are under way. Although there was an increase in equipment-related CIs during the first 6 years of reporting, no significant time correlation could be established.

The second largest proportion of CIs in our data set refers to clinical practice. Although no agreed-upon classification system exists so far [14], it has to be acknowledged that this category comprises a heterogeneous group of CIs such as pressure sores, handling of specimens, infection control, and airway management. Medical complications after interventions, also termed procedural complications [14] (for example, brachial plexus injury and pneumothorax); diagnostic errors; and omission of required investigations (for example, blood glucose monitoring and chest x-ray) were all listed under clinical practice. Failures to comply with management or diagnostic guidelines (for example, review of chest x-ray after line insertion or no monitoring of depth of neuromuscular blockade in patients receiving neuromuscular blocking agents) were summarized under the heading of poor practice. CIs relating to clinical practice can potentially be addressed by continuous education of medical and nursing staff as well as by providing easy access to guidelines and unit policies. Further studies will be required to assess the impact of the introduction of a dedicated practice educator on the incidence of these CIs.

Medication errors comprise a major proportion of CIs in critical care and account for 78% of serious medical errors [3]. Incidents involving pharmaceuticals can be defined as any error in the medication process, including prescription, transcription, preparation, dispensation, and administration of drugs. The consequences of drug-related errors such as increased mortality, morbidity, and hospital length of stay have been outlined in two publications [1,15]. Our data confirm that medication errors are common in the ICU. It remains unclear, however, to what degree they contribute to mortality and morbidity in our patient cohort. Standardized drug concentrations and routine checks at shift change are recognized strategies to reduce the number of medication errors and represent standard practice in our ICU. All prescription charts are checked daily by a dedicated intensive care pharmacist to minimize prescription errors. This might explain why in this study - in contrast to other studies [11,16,17] - medication errors were not the commonest reported type of CI.

The fourth major group (18.9%) of incidents consisted of administrative issues and was in nearly two thirds of cases (63.4%) related to staffing, shortage of beds, and delays in admission and discharge. Two studies indicate that a staffing shortage poses a major risk to patient safety [18,19], although adjustment for confounding factors may weaken the evidence [20]. In particular, prevention of ventilation-associated complications such as unplanned extubations, ventilator-associated pneumonia, and non-compliance with lung-protective tidal volumes requires high nurse-to-patient ratios [21,22]. Current recommendations, therefore, claim one nurse for each patient as the gold standard in critical care nursing [23]. Local policies suggest closure of ICU beds if a one-to-one nurse-to-patient ratio is not achieved. Shortage of ICU beds, however, represents another major proportion of CIs. This observation may reflect the nationwide situation since the UK has the lowest ratio of ICU beds per 100,000 population in Europe [24]. Reporting of staffing and bed shortage and delayed discharges and admissions may help to quantify the actual infrastructural requirements of local and regional ICU services and allow inter-regional and nationwide comparisons.

The remainder of incidents included injuries and safety hazards (7.2%), including slips, trips, and falls, and environmental issues (2%) related to alarms, water supply and pipes, air conditioning, doors, and waste disposal; together, these represented only a small proportion of all incidents reported. It worth noting that a high number of staff injuries were reported. It is likely that staff injuries, mainly needle-stick injuries, are reported more frequently because of medico-legal consequences for the staff member if the incident is not documented. Hence, it seems likely that under-reporting occurs less often when staff injury is involved.

Our study has several limitations. Under-reporting is a common problem associated with documentation of CIs [25]. The fear of reprisal appears to be the main cause for reluctance to report CIs [26]. In an attempt to minimize under-reporting, we have deliberately chosen to retain paper-based CI reporting, which protects anonymity. We did not find an increase in the numbers of CIs reported during the study period. This could be due to either a lack of under-reporting or an insufficient endeavor to quantify under-reporting. A questionnaire survey performed on our unit revealed that less than a quarter of staff have reported a CI within the last month and that the average time that had passed since the last CI report was 24 weeks. We have aimed to reduce under-reporting by promoting a non-punitive and non-confrontational atmosphere. This process included the introduction of multi-disciplinary safety meetings to facilitate communication among doctors, nurses, and other staff to create a culture of 'speaking up'. The first results of these changes are evidenced by an increased reporting rate in our ICU compared with that in other departments within this hospital and a higher reporting rate in our ICU compared with that in other ICUs within our region. In addition, the rate of 10 to 15 CI reports per month in our unit is significantly higher than those in previous reports, in which inclusion of 306 health-care facilities led to only 94 reports per year [27].

A recent study investigating the temporal trends in patient harm concluded that harm remains common with little sign of improvement [28]. This indicates that analysis of large CI databases can constitute only the first step in learning and improvement. It remains to be elucidated whether or not interventions implemented after CI analysis, such as additional training for staff, electronic prescribing, or improvement in equipment, lead to increased patient safety in ICUs.

## Conclusions

In summary, our results suggest that analysis of CI types and frequencies is crucial to raise awareness, identify common incidents, and implement measures to avoid them. We have shown that CIs in critical care most commonly involve medication, equipment, and clinical practice. Structural and procedural changes in critical care, including equipment training for staff, continuous medical education, and guidelines for drug administration, are needed, and the effectiveness, efficiency and efficacy of these measures remain to be evaluated in the future.

## Key messages

• Simple anonymous incident reporting systems may improve reporting rates over complex non-anonymous computer-based systems.

• Critical incidents related to equipment, clinical practice, and pharmaceuticals account for more than two thirds of events reported.

• Equipment-related critical incidents remain the largest single group of reported incidents. Training and familiarization programs, together with registers of users, may help to reduce this type of incident.

• Administrative and staffing issues produce a large number of critical incident reports.

• The lack of widely agreed-upon classification systems for critical incident types can hamper comparisons with other studies.

## Abbreviations

CI: critical incident; ICU: intensive care unit.

## Competing interests

The authors declare that they have no competing interests.

## Authors' contributions

IDW collated and analyzed the data and drafted the manuscript. JG collated and analyzed the data. MM performed the statistical analysis and designed the figures. RW managed the original database. All authors read and approved the final manuscript.
